# The associations of CSF ApoE and C1q with Alzheimer's Disease biomarkers

**DOI:** 10.1002/alz70855_096536

**Published:** 2025-12-23

**Authors:** Zuo‐teng Wang, Lan Tan, Yujing Lin

**Affiliations:** ^1^ Qingdao Municipal Hospital, Qingdao, Shandong, China; ^2^ Qingdao Municipal hospital, Qingdao university, Qingdao, Shandong, China; ^3^ Qingdao Municipal Hospital;Shandong Second Medical University, Qingdao, Shandong, China

## Abstract

**Background:**

The roles of complement 1q (C1q) and Apolipoprotein E (ApoE) in driving Alzheimer's disease (AD) progression might be explained by their associations with neuroinflammation and AD pathology which were previously reported. We examined the associations of cerebrospinal fluid (CSF) C1q and ApoE with CSF neuroinflammatory biomarkers and AD core biomarkers, as well as explore whether C1q mediated the associations of CSF ApoE with these biomarkers.

**Method:**

Here, we analyzed CSF proteomics data using two different proteomics datasets—SomaScan(*n* = 579) and multiple reaction monitoring (MRM[*n* = 207]). Linear regression analyses were conducted to explore the association of CSF ApoE and C1q. The mediation model and structural equation model (SEM) were conducted to explore the associations of ApoE and C1q with AD biomarkers.

**Result:**

Multiple linear regression showed that CSF ApoE was positively associated with CSF C1q in total participants and Alzheimer's continuum participants. Mediation analyses indicated that C1q mediated the associations of CSF ApoE with CSF totau tau(T‐tau), phosphorylated tau(*p*‐tau), glial fibrillary acidic protein(sTREM2) and GFAP(glial fibrillary acidic protein) (all the p values <0.05) but not with CSF amyloid‐β and progranulin (PGRN). The SEM yielded similar results.

**Conclusion:**

Our findings suggest that C1q is linked to ApoE and it mediates the associations of ApoE with T‐tau, *p*‐tau, sTREM2, GFAP, indicating C1q association with ApoE might be involved in AD progression.

